# Trait Self-Control Outperforms Trait Fatigue in Predicting MS Patients' Cortical and Perceptual Responses to an Exhaustive Task

**DOI:** 10.1155/2019/8527203

**Published:** 2019-04-24

**Authors:** Wanja Wolff, Julia Schüler, Jonas Hofstetter, Lorena Baumann, Lena Wolf, Christian Dettmers

**Affiliations:** ^1^University of Konstanz, Germany; ^2^University of Bern, Switzerland; ^3^Kliniken Schmieder, Konstanz, Germany

## Abstract

Patients with multiple sclerosis (PwMS) frequently suffer from fatigue, but this debilitating symptom is not yet fully understood. We propose that self-control can be conceptually and mechanistically linked to the fatigue concept and might help explain some of the diversity on how PwMS who suffer from fatigue deal with this symptom. To test this claim, we first assessed how cortical oxygenation and measures of motor and cognitive state fatigue change during a strenuous physical task, and then we tested the predictive validity of trait fatigue and trait self-control in explaining the observed changes. A sample of *N* = 51 PwMS first completed a test battery to collect trait measures of fatigue and self-control. PwMS then performed an isometric hand contraction task at 10% of their maximum voluntary contraction until exhaustion while we repeatedly assessed ratings of perceived cognitive and motor exertion. In addition, we continuously measured oxygenation of the prefrontal cortex (PFC) using functional near-infrared spectroscopy. Linear mixed-effect models revealed significant increases in perceived motor and cognitive exertion, as well as increases in PFC oxygenation. Hierarchical stepwise regression analyses showed that higher trait self-control predicted a less steep increase in PFC oxygenation and perceived cognitive exertion, while trait fatigue did not predict change in any dependent variable. These results provide preliminary evidence for the suggested link between self-control and fatigue. As self-control can be enhanced with training, this finding possibly has important implications for devising nonpharmacological interventions to help patients deal with symptoms of fatigue.

## 1. Introduction

Most patients with multiple sclerosis (PwMS) suffer from fatigue [1], and 40% of PwMS rate fatigue as their most debilitating symptom [2]. Notwithstanding its importance for PwMS, fatigue is still not fully understood [3]. This is at least in part due to the many different facets of the symptom itself. For example, fatigue can be conceptualized as a trait or a state [4]. This implies that patients who suffer from trait fatigue tend to experience more severe state fatigue in fatigue-inducing situations [5]. Further, fatigue can manifest itself in the cognitive and/or the motor domain [6]. Thus, a PwMS might experience fatigue during physical tasks (motor fatigue) but not during tasks that are mentally taxing (cognitive fatigue) or vice versa. Performance fatigability (i.e., decreases in cognitive or motor performance) has been suggested as an objective measure of fatigue [7, 8]. However, a PwMS might show no decrements in overt performance but suffer from substantial perceptions of fatigue (e.g., [9]). In fact, performance fatigability and perception of fatigue are often only weakly correlated [1], implying that behavioral performance is probably not an ideal measure for fatigue and vice versa [10].

One possible explanation for this disconnect is that PwMS have to recruit more cortical resources to compensate for disease-induced structural (e.g., atrophy) and functional (e.g., connectivity) changes in the brain [4, 11, 12]. Particularly, structures in the corticostriatal network have been implicated in fatigue [13]. For example, increased activity in the nonmotor functions of the basal ganglia has been linked to trait fatigue [4] and higher activations of prefrontal cortex (PFC) areas have been interpreted as a marker of fatigue in PwMS [9]. It has been suggested that perception of fatigue might be “due to an imbalance in perception of energetic costs of an action (effort) and benefits of the resulting outcome (reward)” ([11], p. 850). Research on fatigue during and after physical exercise tends to corroborate this. Ratings of perceived exertion (RPE) increase at a higher rate in PwMS compared to a healthy control group during a handgrip task [14], and performance fatigability tends to be higher in MS patients [15, 16]. Importantly, this is not the result of more peripheral fatigue, as measured by twitches evoked using electrical tetanic stimulation: after a fatiguing task, PwMS in fact displayed less peripheral fatigue but more central fatigue [16]. In line with the above, these findings are explained by a “reduced capacity to compensate for disease-induced damage” ([16], p. 125) and the proposed involvement of higher activity in “areas involved in cost-benefit trade-offs” ([17], p. 801).

Importantly, not only are areas in the PFC implicated in determining action costs (i.e., being a marker for fatigue) but the PFC is also the key structure for exerting voluntary control (i.e., continuing with a course of action, even though perceived costs are high; [18]). This act of controlling an impulse, in order to reach a valued outcome has been termed self-control [19, 20], and a multitude of studies has shown that both high trait self-control and high state self-control are conducive to various positive outcomes (e.g., [21]). For example, in order to reach the long-term goal of improved physical fitness, a PwMS must suppress the urge to terminate a strenuous physical task (i.e., continue although she experiences substantial fatigue).

We propose that self-control and fatigue are closely connected on a conceptual level: the application of self-control is aversive [22] and people try to avoid it [23]. Thus, self-control requires mental effort and exertion of effort leads to sensations of fatigue [24]. From this perspective, fatigue might be understood as a sensation that directly signals the costs of the invested mental effort that was needed to perform an act of self-control. The decision to exert mental effort in the service of self-control hinges on an evaluation of the expected value of control (EVC; [25]). Thus, people aim at maximizing returns obtained from exerting control, and when the expected value of control is too low, no (further) control will be applied. If PwMS have to exert more effort for obtaining a certain reward [4], contemporary self-control theories (e.g., [25–28]) would predict that this should skew the EVC in a way that control incurs higher costs per unit of time (e.g., higher perceived fatigue) or that exertion of control is simply not worthwhile (e.g., increased performance fatigability).

Importantly, these similarities between fatigue and self-control do not stop at the conceptual level outlined above. The neuronal networks that have been implicated in fatigue also play a role in self-control [25, 29, 30]. Different parts of the PFC are differentially involved in the application and evaluation of control. Examples are the control of impulses [29, 31], the valuation of prospective effort exertion [32], and the evaluation of a given course of action in competition with an alternative course of action [33]. One example attesting to the importance of these control relevant areas in regard to fatigue is the higher fatigue levels experienced by patients with lesions in the ventromedial PFC compared to patients with other lesions or the fatigue levels of healthy controls [34]. A large body of research has found increases in PFC activation during self-control-demanding tasks (e.g., [24, 35]). As such tasks frequently tend to be fatiguing, it is difficult to assess whether PFC activation reflects fatigue, the exertion of self-control, or possibly both. Evidence for a self-control interpretation comes from a recent study that showed that a psychological strategy that reduced task-imposed self-control demands led to a less pronounced increase in PFC activation during a strenuous task, while not affecting ratings of perceived exertion [35].

## 2. The Present Study

The goal of the present study was to monitor changes in perceived cognitive and motor exertion and changes in PFC oxygenation that occur while PwMS performed a strenuous physical task and to assess whether such changes can be predicted by measures of trait fatigue and/or trait self-control. We chose a strenuous physical task because such tasks can induce both motor and cognitive fatigue (e.g., [36]) and require self-control (e.g., [37]). As we were interested in changes that occur during strenuous physical activity, we used functional near-infrared spectroscopy (fNIRS) to monitor changes in PFC oxygenation. fNIRS has sufficient temporal resolution and is relatively robust towards motion artifacts and has therefore been recommended and employed for such tasks [35, 38, 39].

As a first research question, we were interested in changes in fatigue-relevant measures that occur during a strenuous physical task. We expected to observe an increase in motor exertion, cognitive exertion, and PFC oxygenation as the task got more fatiguing. Second, there is a gap in the literature as to whether possible changes in these measures reflect increasing fatigue and/or increasing self-control exertion. To address this gap, we tested which trait measure is more successful in accounting for the rate of change that would be observed in perceived motor exertion, perceived cognitive exertion, and PFC activation.

## 3. Methods

### 3.1. Participants

Fifty-one patients (78.4% = female) who were admitted for rehabilitation of MS at Kliniken Schmieder (Germany) took part in the study (age: *M* = 50.12 ± 8.14 years). Most patients were diagnosed with relapsing remitting MS (52.9%), followed by secondary-progressive MS (35.3%) and primary-progressive MS (11.8%). The average time since their MS diagnosis was 17.94 (±8.40) years. The average BDI score was 8.26 (±6.10) indicating clinically unremarkable to mild depression symptoms in the overall sample. The average Extended Disability Status Scale (EDSS) score was 4.6 (±1.48). The EDSS is a scale representing disability due to MS. Zero means no symptoms at all, and ten means death due to MS. The range between 3 and 6 covers moderate disability. Twelve PwMS relied on either a wheelchair (*n* = 3; EDSS ≥ 7) or a mobility aid (*n* = 9; EDSS ≥ 6 and <7) due to impaired lower limb functionality. As PwMS were asked to perform a strenuous handgrip task, only PwMS with sufficient upper limb functionality were eligible for participation. All recruited PwMS participated in tailored physical activity programs as part of their rehabilitation treatment at Kliniken Schmieder, Konstanz, as regular exercise training has been associated with moderate reductions in fatigue among PwMS [40]. Study participation was voluntary and without compensation. The procedure was approved by the Ethics Committee at the University of Konstanz (approval #23/2017).

### 3.2. Measures and Apparatus

#### 3.2.1. The Strenuous Physical Task

The strenuous physical task was set up as a time-to-failure (TTF) task, in order to ensure that each PwMS was fully exerted upon task completion. For this, we used a static hand dynamometer that allows the measurement of hand grip force (HD-BTA; Vernier Software & Technology, Beaverton, OR, USA). In this isometric contraction task (ICT), PwMS were asked to maintain a target grip force for as long as possible. To make task demands comparable between PwMS of different strength, the grip force to be produced represented 10% of ones' maximum voluntary contraction (MVC). To get a valid estimate of MVC, PwMS were asked to produce their maximum grip force three times, separated by breaks of 20 seconds. During the MVC assessment, they were verbally encouraged by the experimenter according to a standardized study protocol. The highest produced force reading was then used as a measure of MVC. MVC measurement and the ICT were performed with the dominant hand, adhering to a standardized grip position. During the ICT, the target grip force was displayed as a horizontal line on a computer screen and the produced force was continuously displayed on the same screen. The task was terminated when PwMS failed to stay above the target line for too long (3 seconds) or if they terminated the task voluntarily.

#### 3.2.2. Assessment of Cerebral Oxygenation

**Figure 1:**
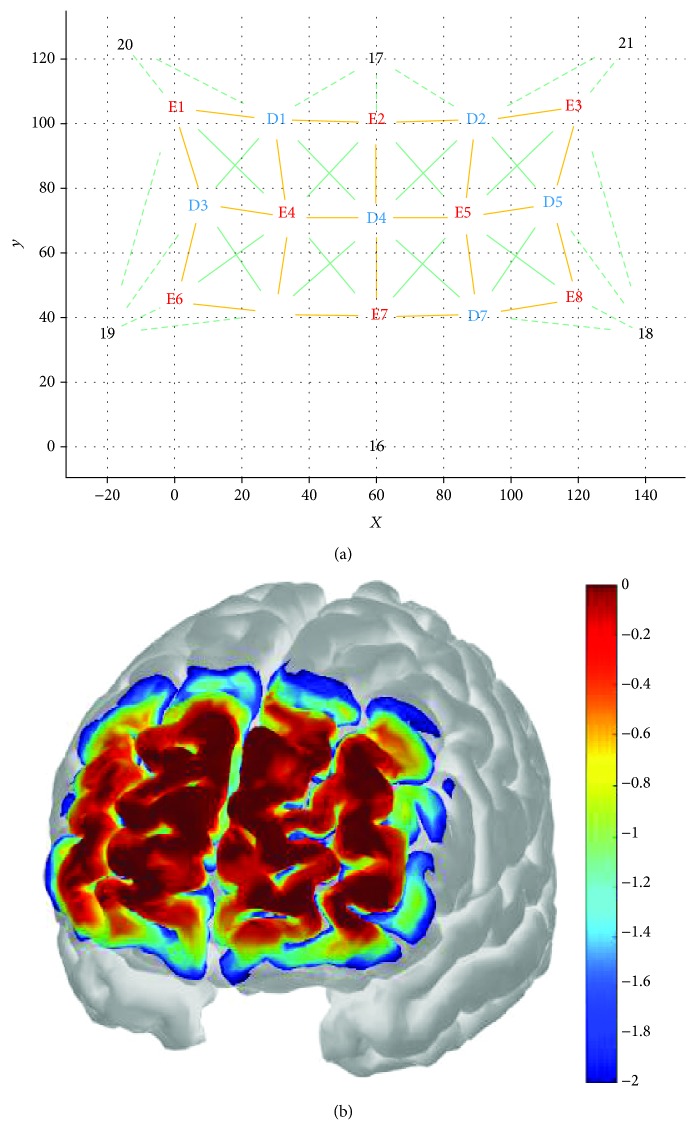
(a) Source-Detector Grid (SD-Grid) as constructed in the SD GUI (AtlasViewer): *X*- and *Y*-axes define a planar space in mm. Yellow lines are channels connecting emitters (in red; E1-E8) with detectors (in blue, D1-D7). Continuous green lines (fixed springs) and channel lines set nonalterable spacing within the probe geometry. Dashed green lines (flexible springs) set alterable spacing within the probe geometry. Springs connect either emitters with detectors or optodes with dummy optodes. Dummy optodes (in black, 16-21) were used to fix the montage to “anchor points” within the 10-5 system for optode placement as specified: 16 at Nz, 17 at AFz, 18 at F7, 19 at F8, 20 at F4, and 21 at F3. Congruence of single emitter (E) and detector (D) positions with the international 10-5 system was maintained for E1 at FFC4, E2 at Fz, E3 at FFC3, E4 at AF4h, E5 at AF3h, E6 at AF8h, E7 at Fpz, E8 at AF7h, D1 at F2, D2 at F1, D3 at AFF6h, D4 at AFz, D5 at AFF5h, D6 at Fp2, and D7 at Fp1. This montage was designed to measure oxygenation concentration at 22 different subareas of the PFC corresponding to the following emitter-detector combinations: E1_D1, E1_D3, E2_D1, E2_D2, E2_D4, E3_D2, E3_D5, E4_D1, E4_D3, E4_D4, E4_D6, E5_D2, E5_D4, E5_D5, E5_D7, E6_D3, E6_D6, E7_D4, E7_D6, E7_D7, E8_D5, and E8_D7. (b) Sensitivity profile as created with AtlasViewer: a Monte Carlo random walk was run with 1*e*^7^ photons (per optode) migrating through a standard atlas.

An 8-emitter + 7-detector multichannel continuous-wave fNIRS imaging system (NIRSport, NIRx Medical Technologies LLC, NY, USA) was used to monitor changes in cerebral oxygenation during the strenuous physical task. NIR light was emitted in two wavelengths (760 nm and 850 nm) at a sampling rate of 7.81 Hz to capture fluctuations in oxygenation. Optodes were fixated in a custom-made stretchy fabric NIRS headband (EASYCAP GmbH, Herrsching, Germany) and positioned onto the forehead site corresponding to the PFC (respective Brodmann area: BA 10). The headband's plug-in mounts enabled measurement of 22 channels with an interoptode distance of approximately 30 mm per emitter-detector combination. Exact relative positions of optodes were measured and reconstructed using the SD-GUI in AtlasViewer v2.3 [41] as shown in Figure 1(a). AtlasViewer was used to register the montage to a standard brain atlas (Colin27) and to obtain optode positions according to the international 5-10 system for optode placement [42]. Registered 5-10 optode positions are specified under Figure 1(b). Probe positioning was standardized by maintaining a 40 mm distance between emitter 7 (E7) and the nasion (Nz) along the dorsoventral axis. The headband was also aligned with the lateral head axis and then attached to the scalp. A calculated sensitivity profile (AtlasViewer v2.3) indicates that the chosen optode placements capture the PFC areas of interest reasonably well (Figure 1(b)).

#### 3.2.3. Ratings of Perceived Exertion (RPE)

While performing the endurance task, an experimenter prompted patients in 30-second intervals to rate their perceived cognitive exertion (RPE_cognitive_) and motor exertion (RPE_motor_) using the category ratio 10 (CR10) scale by Borg [43]. The CR10 scale is “a general intensity scale that can be used to estimate most kinds of perceptual intensities” ([43], p. 15). It can be used in various settings and for different purposes (i.e., studies in medicine and sports). The two scales ranged from 0 (“nothing at all”) to 10 (“maximal”) or 11 (“even more than max”) and were printed on a sheet of paper and were attached to the right corner next to the screen displaying the patient's produced force and target grip force in real-time.

#### 3.2.4. Self-Report Trait Measures

Trait fatigue and self-control were collected with self-report measures. To control for depression, PwMS also completed Beck's Depression Inventory (BDI; [44]), which was used as a control variable in the statistical analyses.

The Fatigue Scale for Motor and Cognitive Functions (FSMC) [6] comprises two subscales which separately assess MS-related cognitive (10 items) and motor (10 items) fatigue. By not relating the phrasing of items to a certain time frame, the FSMC intends to capture stable or trait-like fatigue constructs. Both the cognitive fatigue subscale (Cronbach's *α* = .94, e.g., “When I am experiencing episodes of exhaustion, I lose concentration considerably quicker than I used to”) and the motor fatigue subscale (Cronbach's *α* = .81, e.g., “When I am experiencing episodes of exhaustion, my movements become noticeably clumsier and less coordinated”) are answered on Likert scales (1: does not apply at all; 5: applies completely). Higher values on the FSMC scales indicate higher levels of fatigue.

The SCS-K-D [45] is a German adaptation and short form of the Self-Control Scale (SCS; [21]) and aims at assessing dispositional self-control. The SCS-K-D consists of 13 items (Cronbach's *α* = .79, e.g., “I am good at resisting temptation”) and is answered on Likert scales (1: does not apply at all, 5: applies completely). All items except for items 1, 9, 12, and 13 are inverted before calculating the final score. The lowest and highest possible final scores of 13 and 65 respectively correspond to extreme low levels and extreme high levels of dispositional self-control. High levels on the SCS-K-D reflect higher trait self-control.

### 3.3. Procedure

Prior to their scheduled testing session, PwMS who volunteered to participate filled out an informed consent form and completed the self-report measures. Each testing session was carried out by two researchers who started the session by preparing the fNIRS measurement and setting up the handgrip device system and monitor screen. One experimenter instructed participants about the correct use of the handgrip device, about the task features of the measurement of MVC and the ICT, and about the correct interpretation and use of the CR10 scale. Simultaneously, the second experimenter mounted the fNIRS. For the setup, the experimenter used the NIRStar signal quality indicator (NIRSport, NIRx Medical Technologies LLC, NY, USA) to establish optimal optical contact for probes before task onset. The headband was attached tight-fittingly in order to exert pressure evenly onto the probes. Optode cable bundles were bilaterally deflected and with some leeway attached to patients' collars to minimize any strain or side pull on probes. The montage setup routine included straightening of the probes to retain the probes' perpendicular angles with the scalp, removal of hair right below optode holders, and dimming of ambient light. The fNIRS recording throughout the endurance task was conducted using NIRStar. After the fNIRS setup was complete and satisfying the signal quality was ensured, experimenters provided a demonstration trial in which patients could familiarize themselves with the handgrip device. If patients had no further questions, MVC was determined. Subsequently, ambient light was dimmed and a final fNIRS channel calibration was performed. A 60 sec baseline fNIRS measurement was taken before onset of the handgrip strength endurance task. To ensure precise matching of the handgrip strength endurance task with the fNIRS recording, triggers were set at the start of the fNIRS baseline measurement, at the start of the endurance task, and at the termination of the endurance task. Besides prompting patient RPE ratings, experimenters did not interact with participants during the endurance task and remained outside their field of vision. PwMS' RPE ratings were documented by the experimenters. After PwMS or experimenters had terminated the task, the patients were debriefed on withholding information regarding the study's intent.

### 3.4. Data Analytic Strategy

As the duration of the ICT varied between PwMS and the fatigue-relevant state measures (RPE_cognitive_, RPE_motor_, and cerebral oxygenation) were sampled with different frequencies, these measures were isotime-standardized in 10% increments from 0% to 100% per PwMS (for a similar approach, see [46]). As a first research question, we were interested in changes in fatigue-relevant measures (RPE_cognitive_, RPE_motor_, and cerebral oxygenation) that occur during a strenuous physical task. To test this, we used mixed-effect ANOVAs to test for differences (within factor time to failure: [0-10%], (10-20%],…, (90-100%]) in these measures during the ICT (see also [35, 46]). Our second research question aimed at identifying which trait measure (self-control or fatigue) is a better predictor of the rate of change in the fatigue-relevant dependent state measures (RPE_cognitive_, RPE_motor_, and cerebral oxygenation). To test this, we first calculated three random intercept linear models per PwMS to obtain the rate of change that occurred during the ICT in the three fatigue-relevant state measures (RPE_cognitive_, RPE_motor_, and cerebral oxygenation). The resulting individual regression slopes were used to quantify change over time. We then used hierarchical linear stepwise regression analyses to test which trait measure (motor fatigue, cognitive fatigue, or self-control) would best predict the steepness of the individual regression slopes. To account for individual differences in performance, time to failure in the ICT was included as a regressor in the null model (to account for possible effects of age and disease duration [47], we also performed analyses where we added these variables as regressors into the null model. This did not alter results in a meaningful way, and in the remainder of the paper, we report the analyses without these additional control variables.). BDI, FSMC_motor_, FSMC_cognitive_, and SCS-K-D were then added as regressors to the model. BDI was included to allow for the possibility that changes could be better explained by depressive symptoms. We then evaluated which regressor (if any) would remain as a predictor in the model.

Data analysis was conducted using the statistical software R [48] and JASP (JASP Team, 2018). Mixed-effect ANOVAs were estimated with the lme4 package (version 1.1-1452) using the Satterthwaite approximation for degrees of freedom of lmerTest (version 2.0-3353), and plots were created with ggplot2 (version 2.2.155).

#### 3.4.1. fNIRS Preprocessing

fNIRS data were preprocessed using HOMER2 v2.3 [49]. For each subject, the *enPruneChannels* function was used with the following function arguments to remove channels when the signal was too weak or too strong: dRange(1) = 1*e*^−2^, dRange(2) = 3*e*, and SNRthresh = 2. Then, optical intensity was converted to optical density using the *Intensity to OD* function. To remove motion artifacts, the *Wavelet_Motion_Correction* was run with an IQR of 1.5 [50]. Then, data were low pass filtered (0.2 Hz) and converted to oxy- and deoxyhemoglobin with the modified Beer-Lambert law [51]. For this conversion, differential path length factors were adjusted to patients' mean age [52] and set to 6.8 (for 760 nm) and 5.8 (for 850 nm) [53]. During fNIRS postprocessing, the mean value of a patient's fNIRS baseline measurement was subtracted from all other oxygenation values. Oxygenation values of two PwMS had to be excluded manually for all channels due to baseline shifts. Those baseline shifts were not automatically detected by the *enPruneChannels* function in HOMER2.

## 4. Results

### 4.1. Descriptive Statistics

**Figure 2:**
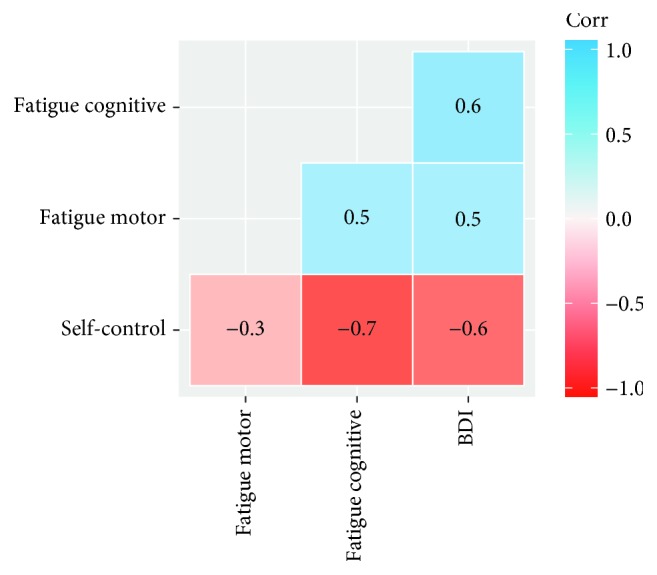
Correlogram of regressor variables.

The average duration on the ICT was 746.39 seconds (SD = 438.63) ranging between 71 and 1823 seconds, which represents a M/SD ratio that is consistent with research using similar tasks [35]. The linear association between regressors can be seen in Figure 2.

### 4.2. Change in Fatigue-Relevant State Variables during Strenuous Physical Activity

**Figure 3:**
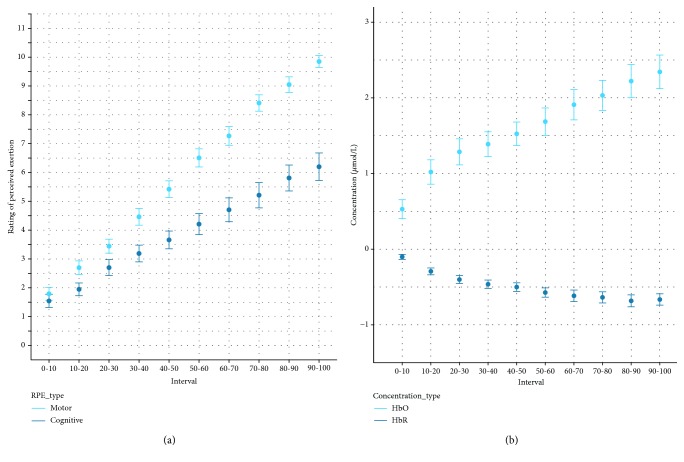
Change in fatigue-relevant state measures (a) and fNIRS signal (b) as a function of isotime in the ICT. Error bars represent standard errors of the mean.

All measured state variables changed in the expected direction: significant main effects of time were observed for RPE_motor_, *F*(9, 421.19) = 332.27, *p* < 0.001, and RPE_cognitive_, *F*(9, 423.56) = 67.65, *p* < 0.001, indicating that the task induced substantial increases in perceived motor and cognitive exertion in PwMS (see Figure 3(a)). In addition, significant main effects of time were observed for oxyhemoglobin, *F*(9, 410.49) = 44.12, *p* < 0.001, and deoxyhemoglobin, *F*(9, 410.42) = 38.711, *p* < 0.001, indicating an increase in cerebral oxygenation that was accompanied by a reduction in deoxyhemoglobin (see Figure 3(b)).

### 4.3. Psychological Predictors of Change

The results of the three stepwise regression analyses for predicting the rate of change in the RPE_motor_, RPE_cognitive_, and oxyhemoglobin are depicted in Table 1. After controlling for TTF, none of the psychological trait variables explained incremental variance in the rate of change in perceived motor exertion during the ICT (all *p* > 0.10). Results were different for regressions on RPE_cognitive_ and cerebral oxygenation: after controlling for TTF, self-control explained incremental variance in these regression analyses. PwMS who reported to be higher in trait self-control experienced a slower increase in perceived cognitive exertion during the ICT, and this was also accompanied by a slower increase in oxyhemoglobin.

## 5. Discussion

The present study showed that not only perception of motor exertion but also perception of cognitive exertion and PFC oxygenation steadily increase during a strenuous physical task in PwMS. Additionally, we found that the increase in perceived cognitive exertion and PFC oxygenation could be predicted by a measure of trait self-control, while measures of trait fatigue (motor and cognitive) were no significant predictors of change in any of these variables.

Our results indicate that a simple strenuous motor task, whose execution outwardly places little cognitive demands on a person, requires cognitive exertion and is accompanied with increased activation in cortical areas that are deemed relevant for fatigue and for self-control. Only trait self-control significantly predicted these changes. This might indicate that the increase in PFC oxygenation and heightened perceptions of mental exertion are more likely to reflect the application of self-control (i.e., the application of effort to continue with the task) than fatigue (i.e., in this context, we interpret the fatigue score as sensation of the costs that are incurred by the task). Consequently, PwMS who were higher in trait self-control experienced a slower increase in perceived cognitive exertion and PFC oxygenation. This is an important contribution to the neuroscientific understanding of fatigue. Specifically, our data indicate that the frequently observed PFC hyperactivations in PwMS might indeed reflect an aspect of fatigue. However, increased PFC activation might not signal the sensation of fatigue but rather the effort that is employed to deal with accruing fatigue. This interpretation can be tentatively interpreted from within the EVC framework [28]. The intensity of the control command rises as one persists in the strenuous task while more intense sensations of motor and cognitive fatigue have to be controlled. People who are better at handling such self-control demands can perform the task with less “cortical costs,” which is then reflected in a less steep increase in PFC activation and RPE_cognitive_. While PFC activation might reflect the effort of dealing with a fatiguing situation, the absence of predictive validity of trait fatigue in our data indicates that the amount of fatigue one experiences seems to be reflected elsewhere. This interpretation is indirectly supported by extant research and theorizing which identify failures in nonmotor functions of the basal ganglia as the most likely source of trait fatigue [4, 13].

### 5.1. Limitations

RPE_motor_ and RPE_cognitive_ are not explicitly designed to capture state fatigue, as, for example, the visual analogue scales used by other researchers are (e.g., [4]). However, research has repeatedly shown that ratings of perceived exertion are sensitive to experimental manipulations of cognitive fatigue (e.g., [54–56]). In the domain of physical exercise, RPE is the standard measure to capture one's state of exertion. RPE is a valid perceptual marker for exercise intensity [57], and it has even been suggested that RPE is the “cardinal exercise stopper” [58]. Therefore, RPE is well suited to assess task-induced exertion fatigue, and this is particularly important for research on strenuous physical exercise in PwMS like the research presented here. Still, we would welcome seeing replications of our research that make use of the state fatigue measures that are commonly used in this line of research.

Our findings pertain to a sample of PwMS and cannot be compared to healthy controls or be generalized to MS patients with lower or higher EDSS. Thus, we cannot assess which of these results are specific to PwMS or would also hold for other patients who suffer from fatigue. However, in supplementary analyses, we additionally controlled for other variables that have been linked to fatigue (i.e., age and disease duration; [47]), and this did not alter results in a meaningful way. This might cautiously be interpreted as an indicator of the robustness of the findings presented here. In the same vein, we cannot compare the observed effects' magnitude with the size of effects in healthy controls. From a perspective of fundamental science, this would be interesting for future research. In the present study, we aimed at adding to our understanding of the temporal dynamics of fatigue in PwMS and the derivation of possible nonpharmacological intervention strategies. For this aim, the lack of comparability with healthy controls is of lesser relevance.

### 5.2. Contribution

The present study is the first to *continuously* monitor changes in variables that are associated with fatigue *while* PwMS performed a strenuous physical task, and it thereby offers a closer look at what PwMS actually *feel* during physical activity and how such perceptions *change* over time. Thus, our research provides a new and facetted angle on fatigue in PwMS, highlighting the cognitive and cerebral costs PwMS incur while performing a strenuous physical task. Most importantly, our findings indicate that the magnitude of these costs varies as a function of self-reported trait self-control. PwMS who are higher in trait self-control seem to incur less costs during strenuous exertion. This finding might offer a promising avenue for nonpharmacological interventions to help PwMS who suffer from fatigue to be more active in spite of their condition: self-control can be trained, and in healthy controls, self-control training can lead to positive outcomes in various domains (for a meta-analysis, see [59]). For PwMS—and other patients who suffer from fatigue—such training might prove to be even more important and helpful. Even though disease-induced structural and functional damages are the probable cause of fatigue, the restriction PwMS experience from this debilitating symptom can likely be modulated by self-control training. We suggest that future nonpharmacological interventions for treating fatigue should directly test this claim and test the effects of self-control interventions on PwMS' fatigue and their capacity to lead a physically more active life.

## Figures and Tables

**Table 1:** Summary of stepwise linear regression analysis for psychological predictors of rate of change in dependent measures.

Measures	Model 0					Model 1				
	*B*	SE *B*	*β*	*R* ^2^	*R* ^2^Adj.	*B*	SE *B*	*β*	*R* ^2^	*R* ^2^Adj.
RPE_motor_				0.626	0.618				—	—
TTF	−0.001	1.143*e*^−4^	−0.791^∗∗^			—	—	—		
SCS	—	—	—			—	—	—		
RPE_cognitive_				0.203	0.186				0.409	0.384
TTF	−5.176*e*^−4^	−1.498*e*^−4^	−0.450^∗∗^			−6.304*e*^−4^	1.333*e*^−4^	−0.548^∗∗^		
SCS	—	—	—			−0.028	0.007	−0.465^∗∗^		
HbO				0.244	0.228				0.367	0.340
TTF	−2.532*e*^−4^	6.497*e*^−5^	−0.494^∗∗^			−2.919*e*^−4^	6.148*e*^−5^	−0.570^∗∗^		
SCS	—	—	—			−0.010	0.003	−0.359^∗∗^		

Notes: ^∗^*p* < 0.05; ^∗∗^*p* < 0.01. RPE_motor_: ratings of perceived motor exertion; RPE_cognitive_: ratings of perceived cognitive exertion; HbO: Concentration of Oxygenated Hemoglobin; TTF: time to failure; SCS: Self-Control Scale.

## Data Availability

The data used to support the findings of this study have not been made available because the patients constituting the sample have not agreed on availability of their data.
